# Nutrition Literacy Level in Bank Employees: The Case of a Large Brazilian Company

**DOI:** 10.3390/nu15102360

**Published:** 2023-05-18

**Authors:** Camila dos Santos Chaves, Juliana Teruel Camargo, Renata Puppin Zandonadi, Eduardo Yoshio Nakano, Verônica Cortez Ginani

**Affiliations:** 1 University of Brasília, Department of Public Health, Brasilia 70910-900, Brazil; milachaves19@gmail.com; 2 National Heart, Lung, and Blood Institute, National Institutes of Health, 10 Center Dr/MSC 1825, Bethesda, MD 20892, USA; juliana.camargo@nih.gov; 3 School of Medicine, University of Kansas Medical Center, 3901 Rainbow Boulevard, Kansas, KS 66160, USA; 4 University of Brasília, Faculty of Health Sciences, Department of Nutrition, Campus Universitario Darcy Ribeiro, Brasilia 70910-900, Brazil; renatapz@unb.br; 5 University of Brasília, Department of Statistics, Brasilia 70910-900, Brazil; nakano@unb.br

**Keywords:** Nutrition Literacy, bank employees, questionnaire online application

## Abstract

Nutrition Literacy (NL) positively impacts diet quality and has the potential to promote health and prevent nutrition-related chronic diseases. Brazil is one of the countries with the highest rates of nutrition-related chronic diseases. Nevertheless, in Brazil, few studies have explored the NL levels of its population. To provide remote access to the Nutrition Literacy Assessment Instrument for Brazilians (NLit-Br) and assess Brazilian bank employees, we conducted a study to estimate the validity of the NLit-Br online and to investigate whether bank employees have an adequate NL level. In the first step, we randomly assigned 21 employees from three financial institution branches to two groups to complete NLit-Br paper and online versions. After an interval period, both groups completed the NLit-Br with an opposite delivery method (paper vs. online). We compared the validity of the digital and paper versions of the NLit-Br by the Intraclass Correlation Coefficient (ICC), and the reliability by Kuder–Richardson formula 20. Second, we evaluated 1174 bank employees using the NLit-Br online version. We found an excellent absolute agreement (ICC ≥ 0.75) between the paper and online versions. The questionnaire had good internal consistency (KR-20 = 0.64). The sample was characterized as mostly male (61.0%), married/cohabitant (73.8%), and white (69.8%), with high household income (85.2%), and graduated or postgraduate (97.4%). The mean age of the population was 42.1 (SD = 7.6) years. Subjects predominantly had possibly inadequate NL (62.3%). The online NLit-Br total score was significantly associated with gender, age, and household income (*p* < 0.05). Women and individuals with higher incomes had a higher degree of NL. Subjects over 50 years old had a lower degree of NL. There was no significant association between the NLit-Br score and the participants’ education. The NLit-Br online is a valid instrument to assess NL remotely. The population studied showed a high prevalence of inadequacy of the NL. Therefore, there is a need for targeted actions to improve the NL of bank employees.

## 1. Introduction

Over 20 million deaths occur yearly from nutrition-related chronic diseases, such as cancer, diabetes, and cardiovascular diseases [1]. In Brazil, the number of deaths recorded in 2019 was 738,371. If we analyze the period from 2000 to 2019, the percentage of premature deaths (those that occurred in individuals aged 30 to 69 years old), although it has decreased, remains alarming. Initially, it was 47.4% and decreased to 41.8% (n = 308,511). Actions aimed at changing this scenario could also benefit populations aged over 70, since the main causes of death and limitations are similar between the two groups. A healthy diet is part of the lifestyle-modifiable behaviors that can prevent chronic diseases [2,3,4,5].

Adopting a healthy diet can be impacted by multiple factors, derived from individual, community, environmental, and structural levels [6]. Among the individual factors, Nutrition Literacy (NL), a set of individual competencies and skills necessary to obtain, understand, and apply nutrition information to make diet decisions [7] is associated with diet quality and health management [8,9].

The NL assessment is a way of associating knowledge about nutrition with appropriate dietary practices to promote positive behavioral changes. The outcomes indicate that knowledge on nutrition is insufficient for adherence to a healthy diet. To consolidate regular healthy eating habits, the individual must have the ability, opportunity, and motivation to identify, understand, interpret, communicate, and use nutritional information in different situations [10,11,12].

The first step is to know the NL and identify the gaps in each population group. In this sense, researchers are conducting NL assessments in groups of parents; teenagers; University students; pregnant women; minority groups such as immigrants; people with NCDs; and breast cancer patients, among others [13,14,15,16,17,18]. Even among these groups, different relationships can be observed between NL and some pre-existing factors. Education and household income, for example, is normally associated proportionally with NL [19].

On the other hand, NL can be a determinant for conditions such as obesity only for some populational groups. A study on the Palestine population revealed a significant association of NL with income and living in a city, relative to a village. However, the researchers did not find a minimal association between diet behavior and Nutrition Literacy, food security and BMI categories [20]. Another study with adolescents in China identified an inverse association between NL and overweight/obesity, especially among those attending senior high schools [21].

NL is an individual factor, but it is impacted by variables associated with a socioeconomic context. These variations must be evaluated according to the reality that different population groups experience. The observed differences regarding the influence of NL on obesity revealed how important it is to assess different populations. Based on the situational diagnosis of the target population, it is possible to plan and develop strategies capable of meeting the specificities of the group and boosting the improvement of the NL level [14].

In Brazil, obesity is a public health problem, mainly for adults. More than half of the adult Brazilian population (60.3%) is overweight. The percentage represents about 82 million people, being more frequent among women (29.5%) than men (21.8%) [22]. In 2019, estimates of obesity prevalence were 20.3%, similar in both sexes [23]. Along with other factors, the numbers presented result in the consolidation of NCDs as the leading cause of mortality in the country. In this sense, in 2016, NCDs accounted for 56% of deaths in the population aged 30 to 69 years [24].

Considering that, in Brazil, this age group is where most of the economically active population is concentrated, actions aimed at changing this scenario are imperative and urgent. The work environment can strongly influence an individual’s eating behavior and health conditions. Similarly, the NL can also be changed by aspects imposed by the work routine and characteristics of the group in question. The predominance of daily intellectual activities and limited food options can motivate the individual to make inappropriate food choices [25]. Therefore, promoting the elevation of NL in economically active populations can be a great challenge.

In the case of bank employees, these characteristics are enhanced. Work-related stress in the financial sector, including banking, is a problem that negatively affects millions of workers. As a negative condition of the work environment, stress presents itself as a health and safety risk worldwide. Its repercussions go beyond work performance and affect workers’ health, social life, and relationships with family and friends [26]. In this way, food can also be the target of imbalance. A study carried out at a banking network in the city of Pelotas (RS, Brazil) identified that, even while understanding aspects that affect blood pressure and cholesterol levels, bank employees had inadequate eating habits (with high consumption of fatty/sweet foods and low consumption of fruits/vegetables) and sedentary behavior [27].

Few studies measured NL in Brazil [28,29,30,31] using three instruments: the Nutrition Literacy Scale (NLS), the Newest Vital Sign (NVS), and the Nutrition Literacy Assessment Instrument (NLit) [28,29,31]. The first comprises 28 items and focuses on reading and comprehending nutrition concepts and recommendations (e.g., calcium is essential for bone health, recommended daily fruits and vegetables portions, and others) [29]. The NVS comprises six items focused on food label interpretation and the mathematical skills to calculate calories and nutrients on the food label, knowledge about the recommended intake of saturated fat and energy, and how to detect allergenic components in a product [28]. The Nlit is validated and available in four languages: English [32], Spanish [15], Italian [33], and Brazilian-Portuguese [31], and includes other crucial concepts for healthy eating, such as knowledge of food groups, portion sizes, and the ability to navigate food marketing. NLit comprises six subscales (64 items): (1) ‘Nutrition & Health’; (2) ‘Energy Sources in Food’; (3) ‘Household Food Measurement’; (4) ‘Food Label and Numeracy’; (5) ‘Food Groups’; (6) ‘Consumer Skills’.

Considering the increasing prevalence of obesity and related problems, such as NCDs, there is a need to expand knowledge on the subject [23]. Another important issue is that no tool is available for remote assessment of individuals about NL. Especially after the COVID-19 pandemic, which required social isolation followed by social distancing, research approaches were reformulated. In addition to meeting the demands of the moment, remote strategies for data collection have numerous advantages. Among them, we can mention the ability to reach a broad and diverse population; convenience for participants in answering the questionnaire virtually; low cost; facilitated data processing; and increased reliability by minimizing the occurrence of missing or inconsistent data using automated features [34,35,36].

Understanding the currently mentioned needs and the possibility of accessing a significant number of subjects, specifically bank employees, this research aimed to (i) validate the NLit-Br for online application and (ii) investigate whether bank employees have an adequate NL level. As a result, it is expected that strategies aimed at food and nutrition education and health appropriate for the population studied will be developed [28,29,37,38].

## 2. Materials and Methods

### 2.1. Study Design

This cross-sectional study was performed in a Brazilian banking institution from the Federal District (FD), between March and September 2020. The Institutional Review Board of the University of Brasilia approved this study (CAAE n 22296019.5.0000.0030). All participants signed informed consent according to institutional guidelines.

### 2.2. Study Participants

During the study period, the FD had 16 banking institutions registered and regulated by the Brazilian Central Bank. The bank studied is one of the largest FD banking institutions, characterized as a mixed capital company in which the Brazilian government holds most of the shares.

The total number of employees active, in 2020, in the FD was 11,930. The study had two inclusion criteria: (1) to be an effective and active employee of one of the participating institutions, and (2) aged ≥ 18 years old. The exclusion criteria included: (1) insufficient visual acuity to read the assessment instrument; (2) employees of outsourced companies, linked to other activities such as cleaning and surveillance; and (3) interns.

The research team requested authorization from the company’s human resources sector to access employees’ emails and send the study form. After approval was received, the human resource sector provided the emails, and we could then follow up with the research.

### 2.3. Nutrition Literacy Assessment Instrument for the Brazilian population (NLit-Br)

The NLit-Br is a previously validated [31] quantitative tool with 64 items, divided into five subscales (nutrition and health, energy sources in food, household food measurement, food label and numeracy, food groups, and consumer skills) to generate a global score for Nutrition Literacy ranging from 0 to 64. The interpretation of the NLit-Br scores was: ≤44 “likelihood of poor Nutrition Literacy”, 45–57 “possibility of poor Nutrition Literacy”, and ≥58 “likelihood of good Nutrition Literacy” [31]. The Nlit-Br was previously validated for application in person, using paper-based questionnaires. Therefore, we performed the tool validation for application online.

### 2.4. Online Version Validation

We performed this step with a convenience sample of 30 adults employed at the same financial institution. We randomly assigned participants into 2 groups. Group 1 (n = 15) completed the paper version followed by the digital version, and group 2 (n = 15) completed the digital version, followed by the paper version. Each participant had a break of 2 h before completing the second version of the questionnaire. The participants did not know that they would answer the questionnaire twice.

### 2.5. Socio-Demographic Questionnaire

All participants answered a structured questionnaire with socio-demographic information to characterize the sample. The socio-demographic questions included education (less than high school, high school graduate, undergraduate, and graduate), household income (Brazilian minimum wages), age, sex, marital status, and race/ethnicity (white, black, brown, yellow, and indigenous) [22,23].

### 2.6. Data Collection and Statistical Analysis

Descriptive statistics, such as mean, standard deviations, and percentages were used to describe the sample. We compared the validity of the digital and paper version of the NLit-Br by the Intraclass Correlation Coefficient (ICC) and the reliability by the Kuder–Richardson formula 20. We considered ICC ≥ 0.6 as a good agreement and ≥0.75 as an excellent agreement [39]. We performed all statistical analyses using IBM SPSS Statistics for Windows, version 22.0. Armonk, NY, USA: IBM Corp.

After validating the tool (NLit-Br) for online use, bank employees received a Microsoft Forms® link that allowed access to the same questionnaire in their corporate email. To ensure that all questions were answered, a feature was used that prevents progression to the next question before the current question is answered. The answers were automatically transferred to a database and linked to Microsoft Forms^®^, providing reliability and facilitating their analysis.

Data were presented using descriptive statistics, such as tables of frequencies, means, median, and standard deviations. The responsiveness of the questionnaire was assessed by floor and ceiling effects. The floor effect is observed when NLit-Br and its domains have a score equal to zero. The ceiling effect occurs when the instrument and its domains reach maximum values [40]. The Kuder–Richardson coefficient (KR-20) was used to assess the internal consistency of NLit-Br. KR-20 > 0.7 is considered good internal consistency [41].

To assess the association between NL and sex, age, income, and education, Student’s *t*-test and Analysis of Variance (ANOVA) with Tukey’s post-test were used. All tests performed considered bilateral hypotheses and a significance level of 5%.

## 3. Results

### 3.1. Validation of the Online Version of the Nutrition Literacy Assessment Instrument for the Brazilian Population (NLit-Br)

Initially, we recruited 30 participants for this first step of the study, but we had nine losses due to incomplete paper questionnaires (n = seven) or withdrawal due to questionnaire length (n = two). As a result, a sample of 21 participants, group 1 (n = 11) and group 2 (n = 10), were eligible for the instrument analysis. On average, participants completed the paper NLit-Br in 18 min and the digital NLit-Br in 20 min. The overall sample of the digital and paper versions of NLit-Br resulted in an excellent agreement (ICC = 0.83). For the subscale scores, food labels and numeracy had an excellent agreement level (ICC = 0.84), and the subscale consumer skills had a lower agreement level (ICC = 0.58) (Table 1).

### 3.2. Assessment of the Degree of NL and Its Association with Socioeconomic and Demographic Factors, Physical Activity, and NCDs in Bank Employees

#### 3.2.1. Socio-Demographic Characteristics

Of 11,930 bank employees working in the FD, 1174 (10%) participated in the study: 39% (n = 458) female and 61% (n = 716) male. Refusals and vacations represented non-participation. The age of our sample ranged from 24 to 68 years, with a mean age of 42 ± 6 y/o. The age group between 40 and 50 years was the most represented. The socioeconomic and demographic characteristics of bank employees are shown in Table 2.

Almost 74% (n = 866) of the participants were married. Regarding monthly household income, most participants (85%; n = 1000) had an income above seven minimum wages (MW), about USD 1390 (one thousand three hundred and ninety dollars equivalent to 7 MW). Other variables evaluated were a) color/race, in which 70% (n = 820) of the population identified themselves as white; and b) education, noting that 74% (n = 870) of the sample had graduated and only 3% (n = 31) had completed high school. Bank employees located in strategic units, that is, linked to directorships, were the most represented (72%; n = 850). As for working time at the institution, 82% (n = 960) of the participants reported working at the bank for more than 10 years.

#### 3.2.2. Nutrition Literacy

When assessing the degree of NL among bank employees, 62.3% (n = 731) of the subjects reached a possibly inadequate level of NL. Only 36.3% (n = 426) were classified as adequate and 1.4% (n = 17) had the lowest score in the category (≤44 points), classified as inadequate NL.

Table 3 presents the NLit-Br scores by domains, responsiveness, and the instrument’s internal consistency assessment data. The mean total score of the NL was 57.72 (SD = 3.91) for a total of 64 items. The result suggests a inadequate level of NL among the population studied. Regarding the domains, it was observed that bank employees had better knowledge concerning the “Nutrition and Health” domain, with a mean score of 9.36 (SD = 0.79). The lowest performance was in the “Home Measures” domain, with an average of 6.52 (SD = 1.58) correct answers. Statistical analysis revealed a KR-20 coefficient (0.64) higher than the one considered good (0.6) for the full scale of the NLit-Br. Thus, the questionnaire can be classified as having good internal consistency [41].

The distributions of variables related to the population’s demographic characteristics according to the degree of NL are recorded in Table 4. The total NLit-Br score is positively associated with gender, age, and household income (*p* < 0.05). The NL degree of women (56.28 points) is significantly higher (*p* = 0.00) than that of men (55.37 points). This difference is also observed concerning the domains “Macronutrients” (*p* = 0.00), “Home Measures” (*p* = 0.00), and “Skills as a Consumer” (*p* = 0.00).

In terms of age, there is a significant difference between the degree of NL in the group with participants up to 39 years old and the group over 50 years old (*p* = 0.00). There was no significant difference in the age group from 40 to 50 years old compared to the groups of up to 39 years old and over 50 years old. Participants over 50 years old have a lower NL degree (54.96 points) compared to participants up to 39 years old (56.20 points) and from 40 to 50 years old (55.63 points). We then realized that the higher the age, the lower the degree of NL.

Analyzing the association of age and NL according to the domains, a significant difference was observed in the domains “Food Groups” between the group up to 39 years old and the group over 50 years old; “Food Labels and Numbers” among the groups up to 39 years old with the group of 40 to 50 years old, and the group of up to 39 years old with the group of 50 years old and over.

As for the household income of the participants, it appears that the degree of NL was significantly higher in the group of participants with more than seven minimum salaries (MS) (55.9 points) when compared to participants with up to five (54.42 points). There was no positive association between the NL degree of participants with a household income of five to seven MS. Thus, we can assume that the higher the household income, the greater the degree of NL.

In the domains “Food Labels and Numbers” and “Skills as a Consumer”, we also found a significant association between the group of participants with a household income of up to five MS and the group with more than seven. In this way, we noticed that higher-income participants have more developed understanding and skills regarding food labels, numeracy, and as a consumer.

There was no significant association between the NLit-Br score and the participants’ education. Specifically, in the domain “Skills as a Consumer” it is noted that participants with a graduate degree (Master’s) have a significantly higher score in this skill than participants with high school diplomas (*p* = 0.05).

## 4. Discussion

### 4.1. Validation of the Online Version of the Nutrition Literacy Assessment Instrument for the Brazilian Population (NLit-Br)

The digital and paper NLit-Br have an excellent level of agreement. We observed similar findings from a socio-demographic questionnaire, in which the digital version provided similar-to-superior quality information compared to the paper version. Digital instruments have advantages over paper instruments: the ability to reach a larger population, lower cost, faster data processing, and decreased missing or inconsistent data [42,43,44].

The overall ICC of the digital version was close to the ICC of the original NLit in English. However, we observed differences in the subscales between the instruments. While the tool tested in Brazil reported a higher ICC for Food Labels and Numeracy, the English version had a higher ICC level for Energy Sources in Food. The differences in subscales’ reliability among nations highlight the importance of environmental and cultural context when exploring nutrition knowledge and skills.

### 4.2. Assessment of the Degree of NL and Its Association with Socioeconomic and Demographic Factors, Physical Activity, and NCDs in Bank Employees

The participation of 1174 (10%) banking employees, working in the FD, was considered good for this study. This can be attributed to the high level of education of the sample, as well as the fact that the questionnaire was answered online with a guarantee of confidentiality of the information. The application of the online version through the Microsoft Forms corporate platform made it possible to disseminate the questionnaire in various sectors of the bank in the region, especially during a pandemic, when most employees were working from home. In addition, the participants could choose the best time to answer the questionnaire (during the work shift), not jeopardizing the undertaking of their tasks.

Men represented (61%) of the participants, revealing a different profile compared to the Brazilian population. According to data released in 2021, by the Continuous National Household Sample Survey—Continuous PNAD, it is known that the Brazilian population from 2012 to 2016 was composed of 48.5% men and 51.5% women. Notably, the number of women is greater after age 25 [45]. The result goes against a trend that has been widely debated concerning banking governance, which is gender diversity. In this sense, studies reveal that there are advantages to the presence of women in banking environments in different administrative areas. Therefore, it is interesting for these institutions to invest in hiring women [46,47].

Both men (80%, n = 572) and women (64%, n = 294) were mostly married/cohabiting. These data are similar to those found in the study by Sanchi and Borges (2019), with bank employees in Rio Grande do Sul (Brazil). Among women, there was a higher proportion of single (22%, n = 99) and divorced (13%, n = 58) than men. Women’s careers are in a ascendancy. However, gender inequalities in labor relations still afflict families in general. Considering the banking universe, gender inequities can negatively stand out. It is observed that the abdication of family routines for successful careers is a constant in the profession. Perhaps for this reason, the lowest female representation and the highest proportion of single and divorced women among the group were detected [48,49,50].

Regarding color, in 2016, there was an increase in the Brazilian population declared to be black (14.9%) and brown (6.6%) compared to 2012 [51]. While the majority of the resident population was brown (46.7% ), 8.2% were black and white (44.2%). Specifically in the Midwest region, 55.3% of the population was brown, 37.0% white, and 6.9% black. Unlike the population of this study, in which 70% of bank employees declared themselves as white, there was no difference in the proportion between men and women. Once again, unequal relations were observed in the evaluated scenario. Despite constituting the majority of the workforce in the country, there is an underutilization of the workforce that comprises people of color, who are black and mixed race, even when considering only the level of education. The phenomenon occurs even among individuals with complete higher education [25].

The sample of bank employees analyzed is a relatively young population. Most are between 24 and 50 y/o, have a high monthly household income (over 7 MW), and a high level of education (graduated) for both genders. Similar to those found in the study by Sanchi and Borges [27], the sample presented household income and an educational level higher than the general Brazilian population.

In 2020, the FD (Brazil) population nominal monthly per capita income ranged from 2 to 4 MW [52]. The usual average real income from work is USD 4.57 (four dollars and fifty-seven cents). The values mentioned are high compared to other states in Brazil and, even so, they are still below the average income found among the bank employees studied. The FD is considered the fifth region in the country, with the highest number of workers (428,785) in public administration, defense, and social security. It thus guarantees a high standard of living for its population even during the crisis resulting from the COVID-19 pandemic [45].

As for the level of NL, it was expected that the largest proportion of bank employees would have adequate nutritional literacy, given their high schooling and higher social class. Upper-middle-class individuals are generally more interested in healthy eating and more knowledgeable about nutrition. In addition, people with a higher level of education would have more numeracy skills and an understanding of NL [53].

However, the results of this study showed that the majority of bank employees, 62.3% (n = 731), reached a possibly inadequate level of NL. This result is similar to the study by Gibbs et al. (2018) [15]. Their research was performed with a Spanish-speaking Latino population in the Midwest of the United States. The mean NLit-S score obtained by the population studied by the authors was 46.6 points.

Regarding the dimensions evaluated in the NLit-Br, it was observed that the domain of “Nutrition and Health” presented a higher performance from the participants (9.36 ± 0.79 points). On the other hand, the domain “Skills as a Consumer” (8.23 ± 0.25 points) and the domain “Home Measures” (6.52 ± 1.58 points) presented the lowest performances.

It is suggested that bank employees are more able to identify and understand information about food sources and examples of foods that make up a healthy diet to prevent and control NCDs. In addition, they have a greater ability to make adequate food choices from a nutritional point of view among similar food options, but difficulty in identifying adequate food portions sizes according to nutritional recommendations.

Current studies have shown that the consumer’s ability to identify and choose sources of macronutrients among similar food product options, considering food marketing, are more critical skills in choosing a healthy diet than the ability to read food labels [32], considering that the NL encompasses knowledge, skills, and behaviors of individuals related to food [8,54,55].

The degree of NL among women was significantly higher than among men (*p* = 0.00). Similar data were found in the study by Kalkan (2019) [56]. The researcher assessed nutritional knowledge and its association with the eating habits of university students in Turkey. In addition to the Turkish women having greater nutritional knowledge, it was found that women had better eating habits than men. The study concluded that nutritional knowledge influences individuals’ eating habits, specifically among women (*p* < 0.05). Brazilian studies point to a greater appreciation of body image by women. This concern with body weight may partially justify the greater interest in aspects involving a healthier diet and, consequently, the greater adequacy of the NL observed [57,58].

Regarding the domains, the higher degree of NL among women in “Macronutrients”, “Home Measures”, and “Skills as a Consumer” may be related to their daily routine. Women are normally responsible for planning household activities and purchasing food. These characteristics may impose the need to develop specific skills. Studies have shown that Brazilian women, especially mothers, are responsible for buying, preparing, and offering food for the family, which is a cultural and social practice [59]. After the insertion of women into the formal job market, activities were overlapped with the accumulation of other daily tasks [60].

Another positive association in this study was between socioeconomic level and the degree of NL, similar to those found in studies on Health Literacy (HL) by Marques, Escarce, and Lemos (2018) [61], and by Ozdemir et al. (2010) [58], with adult primary health care users and patients from a medical clinic in Turkey, respectively.

Income is a determining social factor when assessing the level of NL in specific population groups. People with low economic status need to plan and manage their food intake differently from people in better financial conditions [12]. Jung et al. (2019) [62], in their study of low-income elderly people residing in the state of Alabama (US), found that the inability to buy food, combined with the limited capacity of the elderly for self-care, contributed to the increase in depressive symptoms, resulting in a lower nutritional status.

Age is also a determining factor in developing NL and HL skills. This study found that bank employees aged over 50 have a lower NL score than those aged up to 39. Other research corroborates this finding [63,64]. For example, Grunert, Wills, and Fernandez-Celemin (2010) [64] studied using and understanding nutrition information on food labels among UK consumers and concluded that younger people and people from higher social classes have higher levels of understanding of the nutrition information on food labels. Nutritional knowledge was a significant predictor of this understanding.

Regarding education, studies have shown a strong positive association with HL [61,65]. Sampaio et al. (2015) [66] investigated the factors associated with HL and its relationship with glycemic control in patients with Diabetes Mellitus 2. They showed that the low education level of the population was associated with a lower HL degree. Santos and Portella (2016) [67] found that the population’s low education (up to 4 years of study) limited the HL performance achieved by the elderly.

However, this study did not show a significant association between the NLit-Br score and the participants’ education. The mean NL score was similar at both levels of education. Possibly, there was no important difference between the levels of education, considering that 97.4% of the participants were undergraduates or more.

### 4.3. Limitations

The study has some limitations, such as being a cross-sectional study and targeting a specific social class. The investigated population has characteristics that cannot be expanded in other contexts, such as a high level of education and access to virtual platforms. Considering our sample size (10% return), we cannot generalize our results for all bank employees. However, we anticipated that this study would not represent the national population because it is an exploratory study with a specific sample. In addition, we observed a scarcity of Brazilian studies on the subject. Our study has many strengths that make it an important contribution to the literature. We used a standardized process to test the instrument’s applicability in both a digital and paper version, including an interval period. Moreover, we are the first study to test the validity of the digital application of the NLit. Previous studies that used the NLit instrument did not compare the validity of the digital version.

## 5. Conclusions

This is the first study to test the validity of the digital application of the NLit and confirmed that the digital NLit-Br is a valid instrument to measure Nutrition Literacy, allowing the remote and precise assessment of Nutrition Literacy for Brazilians.

Regarding the employee population of banks located on FD/Brazil, the study highlighted the importance of assessing the degree of NL and associated factors, such as gender, education, and income, in a specific population group. The results can be used to strengthen and implement food and nutrition education actions. Joint strategies from managers and the multidisciplinary health team are needed for educational actions for prevention and health promotion, considering the population profile and NL. Empowering bank employees to care for their health and food can raise the NL level, improving their ability to make healthy food choices, and impacting the prevention and control of NCDs.

Among the different groups of workers, bank employees may be discouraged from correctly using their nutrition knowledge due to the stress they experience daily. Despite having a high level of education, aspects of the professional routine can negatively influence eating behavior. For the group of bank employees, the application of the NL may guide interventions that help improve individuals’ health, according to a particular reality, thus, boosting professional and personal performance and generating a collective benefit.

## Figures and Tables

**Table 1:** NLit-Br paper and digital scores and intraclass correlation coefficient (n = 21).

	Group 1 (n = 11)			Group 2 (n = 10)			Overall (n = 21)		
	Paper	Digital		Paper	Digital		Paper	Digital	
	Mean ± SD	Mean ± SD	ICC	Mean ± SD	Mean ± SD	ICC	Mean ± SD	Mean ± SD	ICC
Nutrition and Health	9.36 ± 0.81	9.27 ± 0.79	0.88	9.50 ± 0.53	9.30 ± 0.82	0.53	9.43 ± 0.68	9.29 ± 0.78	0.75
Energy Sources in Food	8.82 ± 1.08	8.64 ± 1.36	0.86	8.50 ± 1.35	9.00 ± 0.82	0.80	8.67 ± 1.20	8.81 ± 1.12	0.82
Household Food Measurement	6.73 ± 1.01	7.09 ± 1.22	0.66	6.30 ± 2.21	6.10 ± 2.13	0.85	6.52 ± 1.91	6.62 ± 1.75	0.81
Food Label and Numeracy	8.00 ± 1.67	8.55 ± 1.75	0.84	7.00 ± 2.11	8.20 ± 2.04	0.84	7.52 ± 1.91	8.38 ± 1.86	0.84
Food Groups	14.09 ± 1.30	13.73 ± 1.90	0.77	14.10 ± 1.85	15.10 ± 1.29	0.50	14.10 ± 1.55	14.38 ± 1.75	0.70
Consumer Skills	8.27 ± 0.79	8.27 ± 0.90	0.64	7.20 ± 1.48	8.00 ± 0.82	0.50	7.76 ± 1.26	8.14 ± 0.85	0.58
Global Score	55.27 ± 0.79	55.55 ± 4.32	0.89	52.60 ± 5.64	55.70 ± 4.06	0.80	54.00 ± 4.54	55.62 ± 4.09	0.83

SD: Standard Deviation. ICC: Intraclass Correlation Coefficient.

**Table 2:** Socioeconomic and demographic characteristics of research participants, Brasilia-FD, Brazil, 2020 (n = 1174).

Characteristics	Male (n = 716)		Female (n = 458)		Total	
	n	%	n	%	n	%
Marital Status						
Never Been Married	95	13	99	22	194	17
Married/Cohabiting	572	80	294	64	866	73
Widowed	1	0	4	1	5	0
Divorced/Separated	45	6	58	13	103	9
Uninformed	3	0	3	0	6	1
Race						
White	500	70	320	70	820	70
Brown	164	23	114	25	278	24
Black	26	4	11	2	37	3
Asian Descent	18	2	8	2	26	2
Indigenous	2	0	1	0	3	0
Uninformed	6	1	4	1	10	1
Education						
High School or Equivalent	23	3	8	2	31	3
Bachelor’s Degree	116	16	62	14	178	15
Graduate Degree	577	81	388	84	965	82
Age Group						
Up to 39 Years	273	38	198	43	471	40
40 to 50 Years	300	42	205	45	505	43
More Than 50 Years	123	17	46	10	169	14
Uninformed	20	3	9	2	29	3
Household Income						
Up to 5 MW *	34	5	25	5	59	5
5.01 SM to 7 MW *	57	8	58	13	115	10
More Than 7 MW *	625	87	375	82	1000	85

* Minimum wages (MW) = USD 199.

**Table 3:** NLit-Br scores for bank employees, responsiveness, and internal consistency of the questionnaire (Brasilia, FD, Brazil, 2020).

Nutrition Literacy Domains	Number of Items	Mean (SD *)	Median (IQR)	Variation	Floor Effect (%)	Ceiling Effect (%)	Internal Consistency (KR-20)
Nutrition and Health	10	9.4 (0.8)	10 (9–10)	5–10	0	52.3%	0.12
Macronutrients	10	9.2 (1.0)	9 (9–10)	0–10	0.1%	46.3%	0.39
Homemade Measures	9	6.5 (1.5)	7 (6–8)	1–9	0	6.2%	0.30
Food Labels and Numbers	10	8.5 (1.7)	9 (8–10)	1–10	0	32.1%	0.59
Food Groups	16	13.9 (1.4)	14 (13–15)	1–16	0	4.5%	0.38
Skills as a Consumer	9	8.2 (0.9)	8 (8–9)	0–9	0.1%	48.0%	0.34
Total	64	55.7 (3.9)	56 (54–58)	22–63	0	0	0.64

* SD = Standard Deviation.

**Table 4:** Mean (SD) of scale subscores subcategorized by gender, age, household income, and education level (n = 1174).

Characteristics	Nutrition and Health	Macronutrients	Homemade Measures	Food Labels and Numbers	Food Groups	Skills as a Consumer	Total
Gender							
Female	9.3 (0.8) ^a^	9.4 (0.8) ^a^	6.8 (1.3) ^a^	8.5 (1.6) ^a^	13.9 (1.2) ^a^	8.3 (0.8) ^a^	56.3 (3.4) ^a^
Male	9.4 (0.8) ^a^	9.0 (1.1) ^b^	6.3 (1.6) ^b^	8.5 (1.7) ^a^	13.9 (1.4) ^a^	8.2 (1.0) ^b^	55.4 (4.2) ^b^
*p* **	0.260	0.000	0.000	0.676	0.983	0.001	0.260
Age group *							
Up to 39 Years	9.3 (0.8) ^a^	9.3 (1.0) ^a^	6.5 (1.5) ^a^	8.8 (1.5) ^a^	14.1 (1.2) ^a^	8.3 (0.9) ^a^	56.2 (3.6) ^a^
40 to 50 Years	9.4 (0.7) ^a^	9.2 (0.9) ^a^	6.5 (1.5) ^a^	8.4 (1.7) ^b^	13.8(1.5) ^ab^	83 (1.0) ^a^	55.6(4.1) ^ab^
More Than 50 Years	9.4(0.8) ^a^	8.9 (1.2) ^ab^	6.5 (1.5) ^a^	8.3 (1.8) ^b^	13.8 (1.3) ^b^	8.1 (0.9) ^a^	54.9 (3.8) ^b^
*p* ***	0.097	0.002	0.797	0.000	0.010	0.133	0.001
Household income							
Up to 5 MS *	9.2 (1.0) ^a^	9.1 (0.9) ^a^	6.6 (1.4) ^a^	7.9 (2.1) ^a^	13.9 (1.2) ^a^	7.8 (1.4) ^a^	54.4 (4.4) ^a^
5.01 SM to 7 MS *	9.3 (0.7) ^a^	9.0 (1.2) ^a^	6.5 (1.6)^a^	8.1 (1.9) ^ab^	13.8 (1.8) ^a^	8.1 (1.0) ^b^	54.8(4.8) ^ab^
More Than 7 MS *	9.4 (0.8) ^a^	9.2 (1.0) ^a^	6.5 (1.5)^a^	8.6 (1.6) ^b^	13.9 (1.3) ^a^	8.3 (0.9) ^b^	55.9 (3.7) ^b^
*p* ***	0.190	0.192	0.888	0.000	0.614	0.000	0.001
Educational level							
High School or Equivalent	9.4 (0.9) ^a^	9.4 (0.8) ^a^	6.3 (1.6) ^a^	8.1 (1.9) ^a^	13.9 (1.2) ^a^	8.0 (1.1) ^a^	55.1 (4.4) ^a^
Bachelor’s Degree	9.3 (0.8) ^a^	9.0 (1.1) ^a^	6.6 (1.5) ^a^	8.5 (1.6) ^a^	13.9 (1.2) ^a^	8.2 (0.8) ^ab^	55.6 (3.7) ^a^
Graduate Degree	9.4 (0.8) ^a^	9.2 (1.0) ^a^	6.5 (1.5) ^a^	8.5 (1.7) ^a^	13.9 (1.4) ^a^	8.2 (1.0) ^ab^	55.7 (3.9) ^a^
Graduate Degree (Master’s or Doctorate)	9.4 (0.7) ^a^	9.2 (0.9) ^a^	6.5 (1.6) ^a^	8.6 (1.4) ^a^	13.9 (1.2) ^a^	8.5 (0.6) ^b^	56.1 (3.5) ^a^
*p* ***	0.917	0.224	0.712	0.464	0.975	0.048	0.602

* 29 individuals did not provide their age. ** Student’s *t*-test. Groups with the same letters do not differ significantly. *** Anova with Tukey post hoc. ^a,b^ Groups with the same letters do not differ significantly.

## Data Availability

The data presented in this study are openly available in [Institutional repository of the University of Brasilia-UnB] at [https://repositorio.unb.br/handle/10482/42299] (accessed on 12 May 2023).
